# The Genetics and Epigenetics of 22q11.2 Deletion Syndrome

**DOI:** 10.3389/fgene.2019.01365

**Published:** 2020-02-06

**Authors:** Qiumei Du, M. Teresa de la Morena, Nicolai S. C. van Oers

**Affiliations:** ^1^ Department of Immunology, The University of Texas Southwestern Medical Center, Dallas, TX, United States; ^2^ Department of Pediatrics, The University of Washington and Seattle Children’s Hospital, Seattle, WA, United States

**Keywords:** 22q11.2 deletion syndrome, DiGeorge syndrome, *TBX1*, noncoding RNAs, microRNAs, epigenetics, haploinsufficiency

## Abstract

Chromosome 22q11.2 deletion syndrome (22q11.2del) is a complex, multi-organ disorder noted for its varying severity and penetrance among those affected. The clinical problems comprise congenital malformations; cardiac problems including outflow tract defects, hypoplasia of the thymus, hypoparathyroidism, and/or dysmorphic facial features. Additional clinical issues that can appear over time are autoimmunity, renal insufficiency, developmental delay, malignancy and neurological manifestations such as schizophrenia. The majority of individuals with 22q11.2del have a 3 Mb deletion of DNA on chromosome 22, leading to a haploinsufficiency of ~106 genes, which comprise coding RNAs, noncoding RNAs, and pseudogenes. The consequent haploinsufficiency of many of the coding genes are well described, including the key roles of *T-box Transcription Factor 1* (*TBX1*) and *DiGeorge Critical Region 8* (*DGCR8*) in the clinical phenotypes. However, the haploinsufficiency of these genes alone cannot account for the tremendous variation in the severity and penetrance of the clinical complications among those affected. Recent RNA and DNA sequencing approaches are uncovering novel genetic and epigenetic differences among 22q11.2del patients that can influence disease severity. In this review, the role of coding and non-coding genes, including microRNAs (miRNA) and long noncoding RNAs (lncRNAs), will be discussed in relation to their bearing on 22q11.2del with an emphasis on *TBX1*.

## Genetics of 22q11.2 Deletion Syndrome

Chromosome 22q11.2 deletion syndrome (22q11.2del) (OMIM #’s 188400, 192430, 611867) is the most common chromosomal microdeletion reported in humans, affecting ~1/4,000 individuals (Botto et al., 2003; Kobrynski and Sullivan, 2007; Fung et al., 2015; Mcdonald-Mcginn et al., 2015). Screening fetuses for 22q11.2del by prenatal procedures reveals an even higher frequency of ~1/1,000, suggesting a high morbidity *in utero* (Wapner et al., 2012; Grati et al., 2015). The disease is multi-syndromic of contrasting severity and penetrance among those affected. It can include congenital heart disease (CHD; especially conotruncal malformations, tetralogy of Fallot, aortic arch abnormalities, truncus arteriosus, ventricular septal defects and vascular rings) and abnormalities of the palate (clefts and velopharyngeal incompetence) (Kobrynski and Sullivan, 2007; Fung et al., 2015; Guna et al., 2015; Mcdonald-Mcginn et al., 2015; Meechan et al., 2015; Morsheimer et al., 2017; Sullivan, 2019). Hypoplasia of the thymus, hypoparathyroidism, dysmorphic facial features, renal and/or skeletal anomalies are also common (Kobrynski and Sullivan, 2007; Mcdonald-Mcginn et al., 2015; Sullivan, 2019). Those in whom an immune deficiency is identified are often classed as having DiGeorge syndrome (Conley et al., 1979; Kobrynski and Sullivan, 2007; Markert et al., 2007; Marcovecchio et al., 2019). DiGeorge syndrome is first suggested following newborn screens for detecting the levels T-cell receptor excision circles (TRECs) as a measure of T cell output from the thymus (**Table 1**) (Botto et al., 2003; Kobrynski and Sullivan, 2007; Mcdonald-Mcginn et al., 2015). Low TRECs can be an indicator of DiGeorge syndrome, with the diagnosis of 22q11.2del subsequently established by FISH or chromosomal microarray technologies (Kwan et al., 2014; Van Der Spek et al., 2015; Schmid et al., 2017; Ravi et al., 2018). The heterogeneous congenital problems for 22q11.2del patients arise from defective remodeling of the pharyngeal region during embryogenesis (Sullivan, 2004; Bassett et al., 2005; Kobrynski and Sullivan, 2007; Fung et al., 2015; Guna et al., 2015; Mcdonald-Mcginn et al., 2015; Baldini et al., 2016). Impacted is the second heart field and the pharyngeal arch arteries, which form the outgrowth vessels of the heart, as well as the pharyngeal pouches (PP), with the 3^rd^ PP forming the thymic lobes and inferior parathyroids (Lindsay et al., 1999; Jerome and Papaioannou, 2001; Lindsay et al., 2001; Merscher et al., 2001; Xu et al., 2004; Chen et al., 2012; Alfano et al., 2019). Over time, individuals with 22q11.2del often exhibit developmental delay and autoimmune manifestations, with their malignancy risk higher compared to the general population. Autism and autism spectrum, anxiety, attention deficit disorders and psychiatric illnesses like schizophrenia are common (Mcdonald-Mcginn et al., 1999; Kobrynski and Sullivan, 2007; Karayiorgou et al., 2010; Morsheimer et al., 2017; Sullivan, 2019; Zinkstok et al., 2019). Males and females with 22q11.2del are equally affected, regardless of their racial/ethnic grouping (Óskarsdóttir et al., 2005; Kobrynski and Sullivan, 2007; Mcdonald-Mcginn et al., 2015; Kruszka et al., 2017). The least concordant phenotypes occur in children of African descent (Kruszka et al., 2017). Not surprisingly given the numerous organ systems affected, 22q11.2del patients have a diminished life expectancy (Repetto et al., 2014). Mid-aged 22q11.2del individuals have a median life expectancy of 42 years compared to normal sibling controls (60–70 yrs. of age) (Mcdonald-Mcginn et al., 2006; Bassett et al., 2009; Bassett et al., 2011; Repetto et al., 2014). With such a wide gamut of problems, a multi-disciplinary clinical approach is often needed to provide adequate care for children and adolescents with 22q11.2del. This can definitely improve the overall quality of life for 22q11.2del patients, which is lower than normal (Fung et al., 2015; Mcdonald-Mcginn et al., 2015). Yet, health care costs for 22q11.2del patients can reach a staggering $1,000,000 during their first 20 years of life (Brenner et al., 2016).

**Table 1:** Clinical manifestations of 22q11.2 and 22q11.2-like deletion syndromes.

	Chromosomal regions affected			
	22q11.2	10p14-13	4q34.1-q35.2	3p10.3
Frequency	1/4000	>100 individuals	rare	rare
Length of DNA deletion	3 Mb or 1.5 Mb	5 Mb	17.4 Mb	300 kb
**Clinical Phenotype**	% occurrence			
Congenital heart disease (CHD)^a^	75%	82%	15%	yes
Immune deficiency (thymic hypoplasia)^b^	50–70%	17%	nr^d^	yes
Hypocalcemia (hypoparathyroidism)	35%	22%	nr^d^	yes
Dysmorphic craniofacial features	50%	50%	95-99%	yes
Developmental delay	50%	80-90%	10%	yes
Renal anomalies	14%	5%	nr^d^	yes
Skeletal defects	60%	nr^d^	55%	nr^d^
Learning problems	70%	80-99%	65%	yes
Psychiatric disorders^c^	30%	nr^d^	nr^d^	nr^d^
Gastrointestinal abnormalities	30%	nr^d^	nr^d^	nr^d^
Digital malformations (polydactyly)	30%	30-80%	88%	nr^d^

a CHD includes interrupted aortic arches, Tetralogy of Fallot, right subclavian artery defects, ventricular septal defects, pulmonary atresia, and other outflow tract anomalies.

b Immune deficiency is defined with peripheral T cell counts less than 1500 cells/µl.

c Psychiatric disorders can include autism, schizophrenia, seizure disorder.

d nr, not reported.

**Figure 1:**
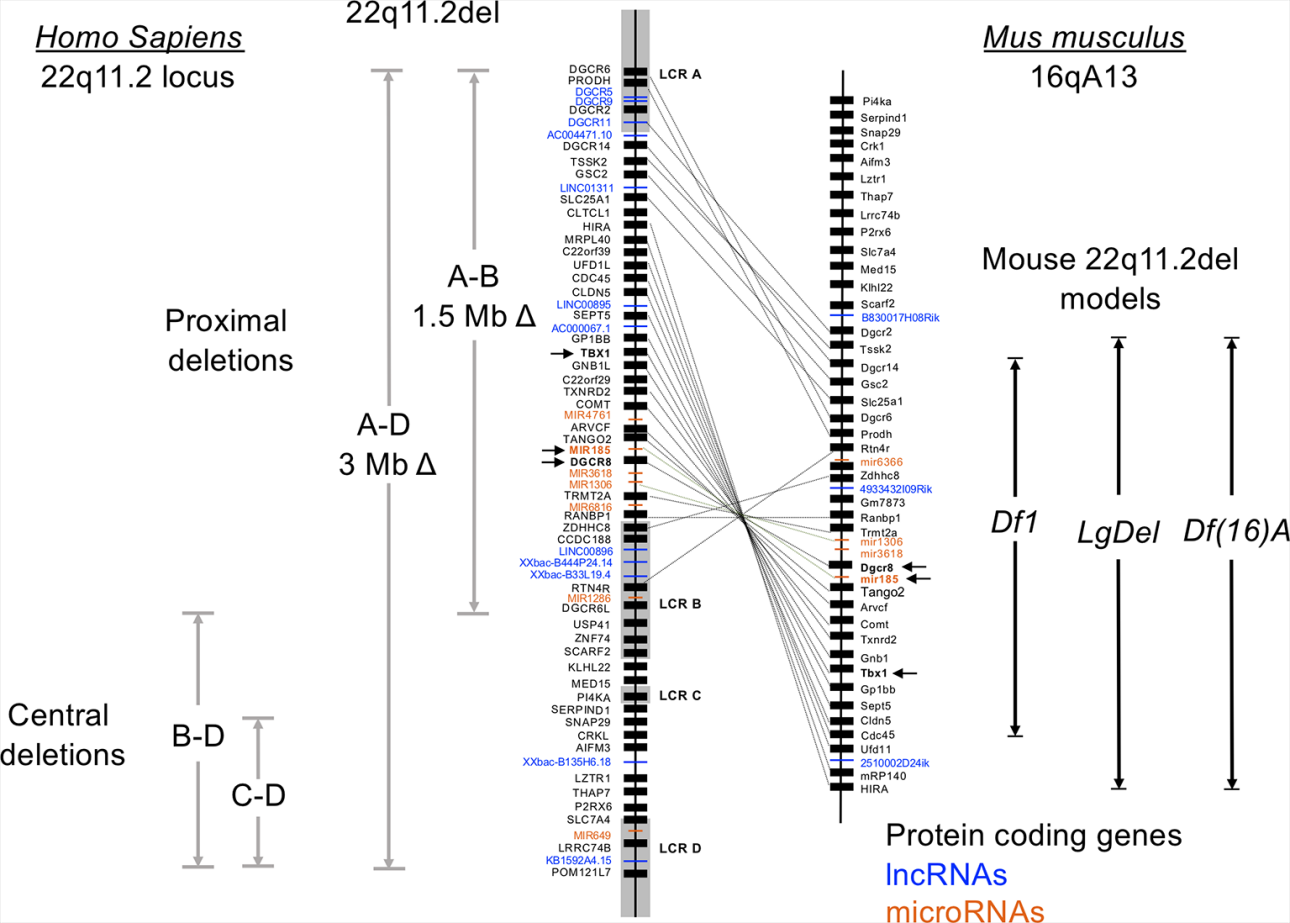
Genetic organization of the human chromosome 22q11.2 locus and synteny on murine chromosome 16. Human chromosome 22 is approximately 51 million base pairs. The region affected by the 22q11.2 chromosomal deletions and duplications spans approximately 4 Mb, with 8 low copy repeats (LCR A-LCR H) causal to this distributed throughout this region. LCR A-LCR D are shown. Recombination of these highly homologous sequences (also called segmental duplications) results in proximal, central, and distal (not shown) deletions affecting both coding and noncoding DNA segments. The proximal deletions are responsible for the classic clinical features of 22q11.2del, which includes DiGeorge syndrome. The location of the coding genes, and noncoding RNAs including miRNAs and lncRNAs, are shown for the proximal region of chromosome 22q11.2. The corresponding genes on the murine locus are connected with lines, which reveals a flipping of the locus and a break containing distinct genes. Three of the more commonly used mouse models of 22q11.2del that replicate many clinical features of 22q11.2del are shown, named as the Df16(A), Df1 and Lgdel lines.

Ninety percent of individuals with 22q11.2del have a 3 Mb microdeletion on chromosome 22, resulting in a hemizygosity of approximately 106 genes (**Figure 1**) (Guna et al., 2015; Mcdonald-Mcginn et al., 2015). Forty-six of these are protein coding, 24 are pseudogenes and the remainder comprise non-coding RNAs; 7 microRNAs (miRNAs), 12 long noncoding RNAs (lncRNAs), 2 small nucleolar RNAs (snoRNAs), and additional undefined transcripts. About 5-8% of 22q11.2del individuals have a smaller, nested deletion of 1.5 Mb (**Figure 1**). This leads to a haploinsufficiency of 30 coding genes, as well as 6 and 9 of the miRNAs and lncRNAs affected with the longer deletion, respectively. Both types of deletions are due to erroneous chromosomal rearrangements during meiosis. These exchanges involve 8 large, paralogous low copy repeats (LCRs), or segmental duplications (A–H) that are distributed along a 5.6 Mb segment of chromosome 22q11.2 (**Figure 1**) (Shaikh et al., 2001; Hwang et al., 2014). DNA recombination of the LCRs, which are 96% sequence identical, during cross-over exchanges results in erroneous deletions or duplications (Shaikh et al., 2000; Shaikh et al., 2001; Bittel et al., 2009). Distributed within or around the LCRs are 8 long intergenic noncoding RNAs (lncRNAs). These lncRNAs contain Translocation Breakpoint Type A (TBTA) sequences with homology to FAM230C, a lncRNA on chromosome 13 (Delihas, 2018). Such lncRNAs may contribute to the deletions *via* the translocation breakpoint sequences. The most frequent deletion in 22q11.2del patients spans LCR A-D (3 Mb), with a less frequent deletion between LCR A-B (1.5 Mb) (**Figure 1**). These are referred to as proximal deletions and are causal to the majority of clinical phenotypes ascribed to 22q11.2del. The actual breakpoint location within the LCR does not have a major role in 22q11.2del phenotypes (Bertini et al., 2017). A rare number of individuals have deletions between LCR B-D or LCR C-D, which are referred to as central deletions. These deletions can also cause CHD and/or neurological abnormalities (Saitta et al., 1999; Burnside, 2015). Even more infrequent are deletions between LCR C-E, LCR D-E, and LCR E-F, although the reported clinical phenotypes are not characteristic of 22q11.2del (Saitta et al., 1999; Shaikh et al., 2007; Burnside, 2015; Guna et al., 2015).

The role of many of the genes whose haploinsufficiency contributes to the congenital malformations, immune system complications, neurological issues and other clinical phenotypes are well-described and discussed in many reviews (Zemble et al., 2010; Bassett et al., 2011; Mcdonald-Mcginn et al., 2015; Meechan et al., 2015; Morsheimer et al., 2017; Morrow et al., 2018; Sullivan, 2019; Zinkstok et al., 2019). Among some of the more well-characterized genes are *T-Box 1 Transcription Factor* (*TBX1*)*, DiGeorge Syndrome Critical Region 8* (*DGCR8*), *Crk-like Adaptor Protein L* (*CRKL*), *Proline Dehydrogenase* (*PRODH*), *Reticulon 4 Receptor* (*RTN4R*), *Zinc-finger DHHC-type Containing 8* (*ZDHHC8*), *Catechol-O-Methyl Transferase* (*COMT*), *Guanine Nucleotide Binding Protein b-polypeptide 1-Like* (*GNB1L*), *Septin 5* (*SEP5*) and *Glycoprotein Ib Platelet Subunit Beta* (*GP1BB*). Yet, the haploinsufficiency of these genes is clearly unable to explain the heterogeneous penetrance and severity of clinical phenotypes that differ from patient to patient. This is even noted among family members with the same mutation in *TBX1* and with genetically identical in-bred strains of mice used as models of 22q11.2del syndrome. In this review, we present recent findings revealing how variations in the expression of *TBX1*, the functions of *DGCR8*, and the miRNAs and long noncoding RNAs within and outside the 22q11.2 locus can influence the severity of the clinical manifestations of 22q11.2del (**Tables 1**–**3**). We also review data identifying gene duplications, CNVs, SNPs, and other chromosomal abnormalities that impact 22q11.2del patients and/or result in overlapping 22q11.2-like phenotypes in some individuals.

**Table 2:** Epigenetic modifiers of 22q11.2 locus: Noncoding RNA regulators and noncoding RNAs.

Type of RNA	RNA transcript affected	Location on 22q11.2	Murine Homologs	Confirmed targets^b^	Tissue Expression	Pathways affected^c^
Modifiers Encoded within 22q11.2del						
Coding	*DiGeorge Syndrome Critical Region 8* (*DGCR8*)	Between LCRA-B	*Dgcr8*	Global mRNAs	Ubiquitous	miRNA biogenesis Alzheimer's disease Schizophrenia
MiRNAs (7)	miR-185	Between LCRA-B	miR-185	*Btk, SERCA2, Camk4, NFAT3c, Mzb1*	Thymus, brain, heart, T and B cells	T and B cell signaling, cardiomyocyte hypertrophy, calcium responses
	miR-4761^a^	Between LCRA-B	none	–	brain	Predicted: Dopamine metabolism and neurotransmitter clearance
	miR-3618	Between LCRA-B 5' UTR of *DGCR8*	none	–	Ubiquitous	Predicted: RNA capping Gene expression
	miR-1306	Between LCRA-B Exon 1 of *DGCR8*	miR-1306	*FBLX5, TGF-beta receptor II*	Ubiquitous	Predicted: mRNA capping and gene expression
	miR-6816^a^	Between LCRA-B	none	–	Not determined	unknown
	miR-1286	Within LCRB	Only in primates^e^	–	Cerebellum	unknown
	miR-649	Within LCRD	miR-649	*MALT1* (NF-kB pathway)	Oral mucosa, muscle	HSV infection susceptibility
	RNA transcript affected	Location on 22q11.2	Highest RPKM (tissue sites)^d^	Dominant Sites of Tissue Expression		Function
LncRNAs (12)	DGCR5	Within LCRA	40 (cerebellum, cortex)	Brain, pancreas, pituitary		Targets miR-1180, miR-23b
	DGCR9	Within LCRA-B	25 (cerebellum)	Brain, pituitary		Cell proliferation and glucose uptake
	DGCR11	Within LCRA-B	1 (Skin)	Brain, skin, blood		Within DGCR2
	AC004471.10	Between LCRA-B	4 (testes)	Brain, muscle, skin, testes		Protein homolog TSSK2
	LINC01311	Between LCRA-B	2.4 (brain, testes)	Ubiquitous		To be determined
	AC000067.2	Between LCRA-B	0.1 (brain)	Arteries, brain		To be determined
	LINC00895	Between LCRA-B	Not reported	Ubiquitous		To be determined
	LINC00896	Between LCRA-B	3.3 (thyroid, pituitary)	Pituitary, thyroid		Regulated by miR-139
	XXbac-B444P24.14 (ENSG00000273139)	Within LCRB	0.8 (pituitary, thyroid)	Ubiquitous		To be determined
	XXbac-B33L19.4 (ENSG00000235578)	Within LCRB	1 (testes)	Testes		To be determined
	XXbac-B135H6.18 (ENSG00000272829)	Between LCRC-D	3 (pituitary)	Brain, muscle, pituitary		To be determined
	KB1592A4.15 (ENSG00000197210.7)	Within LCRD	40 (testes)	Testes		To be determined
SnoRNAs (2)	SNORA15	Between LCRA-B	0.2	Nucleolus		Nucleolar RNA guide for modifying uridine
	SNORA77	Between LCRA-B	5 (brain) 7 (testes)	Nucleolus		Guide for modifying uridine on 18S rRNA
Modifiers Encoded outside the 22q11.2del locus						
Type of RNA	RNA transcript affected	Location	Murine Homologs	Confirmed targets	Tissue expression	Pathways affected^b^
MiRNAs	miR-96a	chr7:129774692-129774769	miR-96a	*TBX1, FOXO1, FOXO3a, RECK, EphrinA5 and SAMD9*	Sensory organs, inner ear, eye, nose	Cilia and hearing
	miR-451a	chr17:28861369-28861400	miR-451a	*TBX1, Mif, c-myc, AKT1, SPARC*	ubiquitous	Wound healing and cellular differentiation

a Read count extremely low in miRbase data set with confirmed biological functions lacking.

b Confirmed targets primarily based on luciferase reporter assays.

c information from published reports, miRNA prediction programs, and Ingenuity Pathway analyses.

d Obtained from uscs genome browser.

e MiR-1286 only present in humans, orangutans, gorillas, and chimpanzees.

**Table 3:** Genetic and epigenetic modifiers of 22q11.2 deletion syndrome with disease connections.

Gene name	Chromosome Location^a^	Function	Human syndromes	Mechanism
Genes on chromosome 22q11.2				
*TBX1*	22q11.2	T-box transcription factor regulates expression of 2000 genes	22q11.2del	Interacts with histone3 methyltransferases, BAF60a, and SMAD1
*DGCR8*	22q11.2	MicroRNA binding protein	22q11.2del	Epigenetically regulates gene expression through miRNAs
*DGCR6*	22q11.2	Regulates neural crest migration	CHD^b^ (with PRODH deletion)	Negatively regulates *TBX1* expression
Morphogens				
Retinoic acid	–	Morphogen involved in embryonic patterning	22q11.2del-like	Complexes retinoic acid receptor to regulate gene expression
Gestational Diabetes	–	Teratogen during pregnancy	22q11.2del-like	Affects multiple developmental processes in the embryo, with the pharyngeal apparatus particularly sensitive
Genes at other chromosomal locations				
*KMT2*	7q36.1	Histone methyltransferase	Kleefstra syndrome 2 (neurodevelopmental disorder)	Interacts with TBX1 and mono-methylates lysine 27 on histone 3
*BAF60a (SMARCD1)*	12q13.2	Component of the SWI-SNF chromatin remodeling complex	–	Component of a chromosome remodeling complex that interacts with TBX1
*MOZ*	8p11.21	Histone acetyl transferase	CHD^b^, intellectual disability, and dysmorphic facial features	positively regulated by RA and, in turn affects TBX1 expression
*SMAD1*	4q31.21	Signaling protein downstream of Bmp4		Interacts with TBX1, which antagonizes its association with SMAD4
*JMJD1C*	10q21.3	Histone demethylase	Rett syndrome and intellectual disability	Demethylates lysine residues on histones
*MINA*	3q11.2	Lysine-Specific Demethylase and Histidyl-Hydroxylase	Preeclampsia	Demethylates lysine residues on histones
*KDM7A*	7q34	Lysine demethylase	–	Demethylates lysine residues on histones
*RREB1*	6p24.3	Zn finger transcription factor	–	Regulates RAS-responsive elements
*SEC24C*	10q22.2	Coat Protein II complex	–	Involved in protein export from ER to Golgi
*GLUT3*	12p13.31	Glucose transporter	-	High affinity interaction with glucose to translocate across the membrane
*KANSL1*	17q21.31	Histone modification	Koolen-De Vries syndrome	Nuclear protein forming part of a complex that has histone acetyltransferase activity
*PAX1*	20p11.22	Transcription factor	Octofaciocervical syndrome	Regulates embryonic tissue patterning
*VEGF*	6p21.1	Vascular endothelial growth factor	Tetralogy of Fallot	Vascular endothelial growth factor supports cell growth
*FGF8*	10q24.32	Fibroblast growth factor	Hypogonadotropic hypogonadism 6	Fibroblast growth factor supports cell growth
*PDGFRα*	4q12	Platelet derived growth factor receptor	–	Regulates neural crest cell development
*SHH*	7q36.3	Sonic hedgehog protein	Holoprosencephaly 3, Microphthalmia with coloboma 5	Regulates Tbx1 expression and vertebrate organogenesis

a Human chromosomal location.

b Congenital heart disease.

## The Genetic and Epigenetic Regulation Coupled to *TBX1* Functions

The congenital malformations associated with 22q11.2del are often linked to the haploinsufficiency of *TBX1* (Lindsay et al., 1999; Jerome and Papaioannou, 2001; Lindsay et al., 2001; Merscher et al., 2001; Zhang and Baldini, 2008; Fulcoli et al., 2016). *TBX1* is expressed at specific regions in the pharyngeal apparatus and in the cardiac progenitors of the second heart field, regulating the expression of nearly 2000 genes (Xu et al., 2004; Chen et al., 2012; Fulcoli et al., 2016). Many excellent reviews detailing the transcriptional role of *TBX1* in the patterning of the pharyngeal arches and pouches have been published (Yagi et al., 2003; Zemble et al., 2010; Bassett et al., 2011; Fung et al., 2015; Mcdonald-Mcginn et al., 2015; Meechan et al., 2015; Baldini et al., 2016; Sullivan, 2019). While one might expect that a simple haploinsufficiency of *TBX1* should consistently lead to similar congenital malformations, this is certainly not what happens. It is becoming apparent that genetic and epigenetic changes, both within and outside chromosome 22q11.2, can dramatically sway the clinical phenotypes of 22q11.2del (Lindsay et al., 1999; Jerome and Papaioannou, 2001; Lindsay et al., 2001; Merscher et al., 2001; Zhang and Baldini, 2008; Fulcoli et al., 2016).

**Figure 2:**
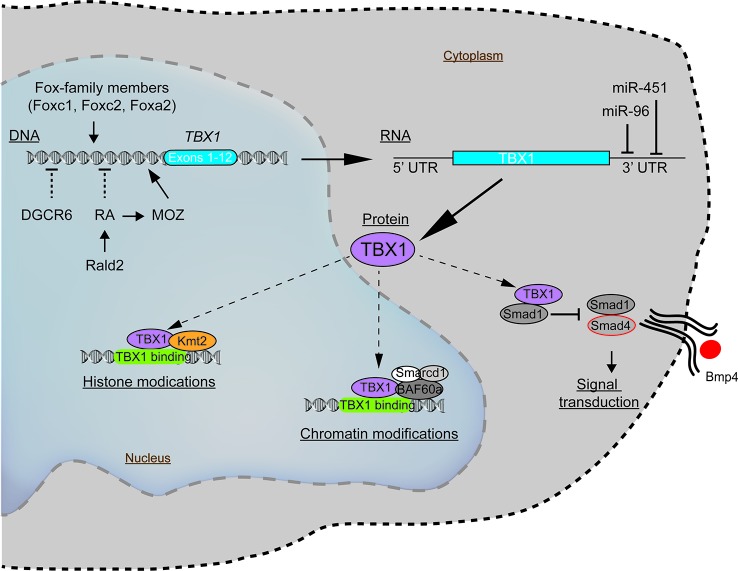
Molecular pathways intersecting with *TBX1* with the potential to modulate the clinical severity and penetrance of 22q11.2del. The expression of *TBX1*, a key gene coupled to the patterning of the pharyngeal apparatus, is positively regulated by members of the Fox-family of transcription factors and MOZ. Negative regulators of TBX1 include the defined teratogens, retinoic acid (RA), gestational diabetes, and miR-96 and miR-451a, the latter two miRNAs that target the 3' untranslated region of TBX1, resulting in its degradation. While most TBX1 resides in the nucleus, wherein it can regulate gene expression, a fraction of TBX1 is localized in the cytosol, where it negatively impacts bone morphogenic protein signaling *via* a complex with SMAD4.

To understand how such genetic and epigenetic factors impact *TBX1* and how this may affect the clinical phenotypes requires some knowledge of how TBX1 functions. Recent evidence indicates that *TBX1* interacts with members of the Histone-lysine N-methyltransferase (KMT2)-family (Fulcoli et al., 2016). The KMT2 enzymes activate transcription by mono-methylating lysine residues on Histone 3 (H3K4) on chromatin (**Figure 2**) (Fulcoli et al., 2016). The TBX1-KMT2 complex regulates the low-level expression of thousands of genes by marking chromatin. In addition, TBX1 interacts with the SWI-SNF-like BAF complex to support chromatin remodeling (**Figure 2**) (Chen et al., 2012). These two types of interactions would suggest that small fluctuations in the levels of TBX1 could modulate the expression of thousands of transcripts, with those impacted likely stochastic from cell to cell. Thus, variations in TBX1 levels could underlie the severity of the malformations in the pharyngeal region. What patient to patient differences exist that could affect *TBX1* expression? One is with *DiGeorge Critical Region 6 (DGCR6*), a gene also encoded on the frequently deleted segment of chromosome 22q11.2. DGCR6 down-regulates *TBX1*, impacting neural crest migration within the pharyngeal region (**Figure 2**, **Table 3**) (Groot et al., 2004; Chakraborty et al., 2012). As neural crest cells establish the vasculature of the pharyngeal arch arteries along with the capsule of the thymus, changes in their location would affect the morphogenesis of this region. While the transcript levels of *DGCR6* as well as its homolog, *DGCR6L* should be reduced 50% due to their respective haploinsufficiency in 22q11.2del patients, their levels are extremely variable in 22q11.2del patients (Chakraborty et al., 2012). Some individuals actually have much higher levels of DGCR6 compared to normal controls (Chakraborty et al., 2012). Such dramatic expression variations in *DGCR6* and *DGCR6L* are epigenetically determined as there is no evidence of maternal or paternal imprinting (Chakraborty et al., 2012). Noteworthy, either deletions or duplications of *DGCR6*, in combination with an adjacent gene, *PRODH*, are linked to CHD (Gao et al., 2015b). These findings suggest that fluctuations in DGCR6 will affect *TBX1* levels, and as a consequence, patterning of the pharyngeal region. While most TBX1 is localized in the nucleus, some is present in the cytosol where a portion is complexed with SMAD1 (Fulcoli et al., 2009). This interaction limits the levels of a SMAD1-SMAD4 complex, preventing effective Bone Morphogenic Protein 4 (BMP4) signaling (**Figure 2**) (Fulcoli et al., 2009). As Bmp4 has a critical role in the septation of the outflow tracts and remodeling of the arch arteries, disruption of its signaling by modulating TBX1 levels is certain to impact these processes (Liu et al., 2004). Bmp4 also regulates early thymus and parathyroid morphogenesis (Gordon et al., 2008). TBX1 interacts with several additional proteins, albeit the role of these complexes are less well-defined (Baldini et al., 2016). In summary, alterations in the levels and/or subcellular distribution of TBX1 will certainly impact the severity of the congenital problems.

The consequence of varying *TBX1* levels in relation to the severity of the congenital malformations in humans is further supported by comparisons with the diverse 22q11.2del mouse models. Multiple mouse lines were originally created with deletions of varying length on chromosome 16 (orthologous to human chromosome 22q11.2) to identify causal genes in the disorder (**Figure 1**) (Jones et al., 1999; Kimber et al., 1999; Lindsay et al., 1999; Puech et al., 2000; Jerome and Papaioannou, 2001; Lindsay et al., 2001; Merscher et al., 2001). Comparing the phenotypes in several such mouse lines confirms that changes in *Tbx1* levels affects the extent and severity of the CHD, thymic hypoplasia, and hypoparathyroidism (Kimber et al., 1999; Lindsay et al., 1999; Puech et al., 2000; Jerome and Papaioannou, 2001; Lindsay and Baldini, 2001; Lindsay et al., 2001; Merscher et al., 2001; Taddei et al., 2001). Yet, relative to humans, mice actually have a lower penetrance of several of the congenital defects in the setting of either the 22q11.2del or just a *Tbx1* haploinsufficiency, most evident with the number of embryos exhibiting a hypoplasia of the thymus. This corresponds to the levels of *TBX1,* since reducing *Tbx1* to 35% normal in the embryos results in a highly penetrant thymic hypoplasia (Zhang and Baldini, 2008). Are there strategies to circumvent the reduced levels of *TBX1*? Yes, as treating pregnant mice with a histone demethylase inhibitor (tranylcypromine) can increase the methylation levels of H3K4 and partially rescue the cardiovascular anomalies in the *Tbx*1-mutant embryos (Fulcoli et al., 2016).

There are several additional regulators of *TBX1* expression that likely have a bearing on 22q11.2del. One is retinoic acid (RA), a metabolite of vitamin A that functions as a natural morphogen involved in the patterning of the 3^rd^, 4^th^, and 6^th^ pharyngeal arch arteries and 3^rd^ PP (**Figure 2**) (Niederreither et al., 2003; Vermot et al., 2003; Cipollone et al., 2006; Voss et al., 2012). Vitamin A deficiency during pregnancy remains a public health issue in developing countries due to its impact on the developing fetus, particularly eye development (Bastos Maia et al., 2019). High levels of RA can act as a teratogen that causes overlapping 22q11.2del-like phenotypes. This was discovered after pregnant mothers were prescribed RA (tretinoin, isotretinoin) as a clinical treatment for acne, with their infants born with 22q11.2-like congenital malformations (Kucera, 1971; Lammer et al., 1985; Lipson et al., 1993; Markert et al., 2007; Mills, 2010; Sacks et al., 2012; Browne et al., 2014). Mechanistically, RA suppresses *TBX1* expression (Roberts et al., 2005; Ryckebusch et al., 2010; Smith and Tallquist, 2010). This was confirmed by using mice created with a haploinsufficiency of *retinaldehyde dehydrogenase 2* (*Rald2*), an enzyme required for RA synthesis. *Rald2^+/-^* embryos produce less RA, increasing the levels of *Tbx1*. In the setting of a *Tbx1* haploinsufficiency, the severity of the heart defects was reduced in the Rald2^+/-^ background (Ryckebusch et al., 2010). RA also acts to epigenetically alter chromatin accessibility by positively regulating the expression of *Monocytic Leukemia Zinc Finger* (*MOZ/Myst3/Kat6a*), a histone acetyltransferase. MOZ, in turn, positively regulates TBX1 levels, with *Moz*-knock-out mice and those with loss-of-function mutations having less *Tbx1*, along with *Tbx2*, *Tbx5*, and *Tbx9* (Roberts et al., 2005; Jenkinson et al., 2007; Voss et al., 2012). In humans, heterozygous MOZ mutations lead to a syndromic disorder comprising CHD, intellectual disability, and dysmorphic facial features (Tham et al., 2015). In summary, too much RA leads to similar clinical phenotypes as 22q11.2del, likely through its ability to reduce the expression of various TBX-family members including *TBX1* (Lindsay et al., 2001; Merscher et al., 2001; Ensenauer et al., 2003; Mokate et al., 2006; Zweier et al., 2007; Zhang and Baldini, 2008).

Gestational diabetes is a distinct teratogen affecting pregnancy, with newborns having congenital features overlapping with 22q11.2del (Kucera, 1971; Mills, 2010; Sacks et al., 2012; Dornemann et al., 2017). In fact, about 18% of infants with a thymic aplasia who did not have deletions on chromosome 22q11.2 and required a thymic tissue transplant were born to mothers with a clinical history of gestational diabetes (Markert et al., 2007). Experimental induction of diabetes in pregnant mice and rats similarly results in a thymic hypoplasia along with intrauterine growth impairment (Padmanabhan and Shafiullah, 2004). So how might elevated blood sugar levels lead to 22q11.2del-like presentations? While this is not yet known, there are some emerging clues. Pregestational diabetes reduces the expression of a regulator of RA, *Cytochrome P450 family 26 subfamily A member 1* (*Cyp26a1*), in the caudal region (tailbud) of developing embryos (Lee et al., 2017). Cyp26a1 catabolizes RA in various tissues to control the levels of this product, and consequently morphogenesis. One speculation is that elevations in blood sugar levels during gestation may increase the levels of RA in the pharyngeal region, thereby diminishing *TBX1* levels. It is also known that gestational diabetes affects histone modifications and DNA methylation patterns in fetal cord blood, implying an epigenetic effect that may contribute to the 22q11.del phenotypes (Haertle et al., 2017). These emerging findings suggest that better control of gestational diabetes may aid in diminishing the severity of certain congenital malformations, particularly for embryos who have 22q11.2del.

## DiGeorge Syndrome Critical Region 8 and microRNA Contributions to 22q11.2del

*DiGeorge Syndrome Critical Region 8 (DGCR8)* is another key gene whose haploinsufficiency in 22q11.2del patients can influence the severity of their clinical problems. It is a nuclear miRNA binding protein required for the biogenesis of microRNAs (miRNAs) (Landthaler et al., 2004; Yi et al., 2009; Schofield et al., 2011). MiRNAs are a family of ~2000 small noncoding RNAs (18-22 nucleotides) that bind to diverse mRNA transcripts, targeting the latter for degradation (Lewis et al., 2005; Grosshans and Filipowicz, 2008; Lodish et al., 2008; Bartel, 2009; Xiao and Rajewsky, 2009; Krol et al., 2010; Salmena et al., 2011; Alles et al., 2019). DGCR8 binds to primary miRNA (pri-miRNA) transcripts in the nucleus in conjunction with DROSHA, an endoribonuclease (Ribonuclease III) that cleaves pri-miRNAs into precursor miRNAs (pre-miRNAs) (Lee et al., 2003; Landthaler et al., 2004). These pre-miRNAs are subsequently exported from the nucleus for further processing into miRNAs. Importantly, such miRNAs regulate cellular homeostasis in almost all tissues, are very stress responsive, and facilitate tissue repair and regeneration (Bartel, 2009; Leung and Sharp, 2010; Mendell and Olson, 2012). Thus, a 50% reduction in *DGCR8* expression due to 22q11.2del would be predicted to modulate the expression of hundreds of miRNAs. Consistent with this, 22q11.2del patients have a miRNA dysregulation in the peripheral blood, with many miRNAs reduced in expression relative to normal controls (**Table 2**) (De La Morena et al., 2013). This dysregulation includes a hypervariable miRNA expression pattern that differs from patient to patient, and interestingly, a coordinated expression of multiple distinct clusters of miRNAs among most 22q11.2del individuals (De La Morena et al., 2013; Sellier et al., 2014). The distinct miRNA clustering may emanate from physiological responses to the clinical problems.

In mouse models, the haploinsufficiency of *Dgcr8* causes a 30-50% reduction in the overall expression of miRNAs screened in neurons (Stark et al., 2008). These miRNA losses worsen as the mice age. One consequence so far described is an abnormal calcium response in the hippocampal pyramidal neurons (CA3-CA1) that leads to premature synaptic neurotransmitter release (Earls et al., 2010; Earls et al., 2012). Such synaptic disruptions are characteristic of schizophrenia, a frequent presentation in the 22q11.2del patients (Karayiorgou et al., 1995; Karayiorgou et al., 2010; Xu et al., 2013). The neurological miRNA deficiencies in the *Dgcr8* ^+/-^ mice are similar in the mouse models of 22q11.2del, establishing that a 50% reduction in *Dgcr8* is key to the miRNA losses (Earls et al., 2012). Interestingly, DGCR8 has functions unrelated to miRNA processing, instead regulating splicing processes in embryonic stem cells, a finding that may reveal more complexities concerning 22q11.2del (Cirera-Salinas et al., 2017).

In addition to *DGCR8*, the frequently deleted segment of chromosome 22q11.2 leads to a haploinsufficiency of 7 miRNAs (**Figure 1**, **Table 2**). Six are clustered near *DGCR8*. MiR-185 has the strongest connection to the clinical phenotypes of 22q11.2del, detected at 0.4 X normal levels in peripheral blood samples (De La Morena et al., 2013). It is expressed in cardiac tissue, immune cells, and in the brain, targeting *Marginal Zone B1 Protein* (*Mzb1*), *Bruton’s Tyrosine Kinase* (*Btk*), *Calmodulin Kinase 4* (*Camk4*), *Sarco/endoplasmic Reticulum Ca^2+-^ATPase* (*Serca2*), and *Nuclear Factor of Activated T cells 3c* (*Nfat3c*) (Earls et al., 2012; Belkaya et al., 2013; Kim et al., 2015). A common theme regarding these miR-185 targets is calcium signaling, central to many cellular processes (**Table 2**). The haploinsufficiency of miR-185 in hippocampal neurons results in premature synaptic transmissions, partly through changes in *Serca2* and *Nfatc* levels, with these genes controlling or being regulated by intracellular calcium changes, respectively (Earls et al., 2012). Moreover, enforced expression of miR-185 in thymocytes attenuates T cell development by a disruption of the T cell receptor controlled calcium response (Belkaya et al., 2013). MiR-185 also controls cardiac hypertrophy by targeting *Nfatc* in myocardial cells (Kim et al., 2015). In B cells, the haploinsufficiency of miR-185 is linked to increased autoantibody production, and this may involve increases in the levels of *Bruton’s tyrosine kinase* (Btk), a target of miR-185 (Belver et al., 2010). Notably, 22q11.2del patients can have clinical phenotypes related to the disruption of all of these pathways; abnormal neurotransmitter release, low T cell output, cardiac hypertrophy, and autoimmunity. Two additional miRNAs, miR-3618 and miR-1306, are located at the 5’ untranslated region and within exon 1 of *DGCR8*, respectively (**Table 2**). While miR-1306 is encoded in most eukaryotic species, miR-3618 is restricted to primates (Merico et al., 2014). MiR-1306 targets *F-box/Leucine rich repeat 5* (*FBLX5*), affecting epithelial to mesenchymal transitions and *TGF beta-receptor 2*, which is involved in the TGF-beta-SMAD4 signaling process (**Figure 2**) (He et al., 2018; Yang et al., 2019). Other haploinsufficient miRNAs include miR-1286, selectively encoded in advanced primates, with *in situ* hybridization revealing its expression in the occipital cortex (Nowakowski et al., 2018). MiR-649, located within the LCRD segment of 22q11.2, targets *MALT1*, a component of a complex of proteins that activate NF-κB in lymphocytes. MiR-649 enhances Herpes Simplex Virus susceptibility, likely through effects on the NF-κB pathway (Zhang et al., 2017). Several miRNA target prediction programs have been applied to the 7 miRNAs encoded on chromosome 22q11.1 to identify linked pathways that relate to clinical phenotypes. An overlapping set of these miRNAs can target *TBX1*, *KAT8 regulatory NSL complex subunit 1* (*KANSL1*), *Glucose Transporter 3* (*GLUT3*, SLC2A3), and *Ras Responsive Element Binding protein 1* (*RREB1*) (Bertini et al., 2017; León et al., 2017). Several of these targets are discussed in a subsequent section in relation to their roles in 22q11.2del. Four of the miRNAs regulate transcripts linked to schizophrenia (Merico et al., 2014).

Interestingly, there are 2 miRNAs encoded on other chromosomes that target *TBX1*. One is miR-96, mutations in which cause a non-syndromic hearing loss in humans (**Figure 2**) (Mencia et al., 2009; Gao et al., 2015a). In a feed-back loop, TBX1 represses miR-96, suggesting that alterations in the levels of *TBX1* may impact hearing loss, consistent with the auditory problems reported for some but not all 22q11.2del patients (Gao et al., 2015a). A second is miR-451, which is involved in wound healing and has been shown to target *TBX1* transcripts (Sun and Jiang, 2018; Munk et al., 2019). Ongoing research efforts to identify the different miRNA targets are uncovering multiple intersecting pathways connected with the developmental and neurological manifestation of 22q11.2del (**Table 2**). The fact that the expression of many distinct miRNAs fluctuate over time and are variable from patient to patient likely explains some of the post-natal complications that differ among 22q11.2del patients (De La Morena et al., 2013). Future directions for such patients may be clinical interventions to improve DGCR8 functions, with recent experiments reporting that protoporphyrins can enhance miRNA biogenesis in DGCR8-haploinsufficient cells (Barr et al., 2015; Sachar et al., 2016).

## Emerging Noncoding RNAs and Their Potential Roles in 22q11.2del

Adding further complexity to 22q11.del are the 12 long noncoding RNAs (lncRNAs) embedded in the frequently deleted segment of chromosome 22q11.2 (**Table 2**). LncRNAs can act as scaffold RNAs, transcriptional assembly hubs, regulators of chromatin accessibility and genome stability (Angrand et al., 2015; Lee et al., 2016; Quinn and Chang, 2016; Du et al., 2019). They exhibit limited evolutionary sequence conservation and are generally expressed at levels much lower than protein coding genes (Cesana et al., 2011; Keniry et al., 2012; Dey et al., 2014; Yang et al., 2014; Zhou et al., 2015). In spite of the accumulating discoveries detailing the functions of numerous lncRNAs, relatively little is known about the 12 that are frequently deleted on chromosome 22q11.2. As the curation of lncRNAs in the human genome is incomplete, additional lncRNAs are likely to be identified on chromosome 22q11.2, while some of those currently compiled may be invalidated. Four lncRNAs, DGCR5, DGCR9, LINC01311, and LINC00896 have high enough RPKM values (Reads per Kilobase of transcripts per Million mapped reads) to suggest functionality, particularly as each is expressed in the biologically relevant tissue (**Table 2**). Findings to date suggest that several of these lncRNAs could affect the clinical presentations of 22q11.2del, but this is only inferred based on what is known about these lncRNAs in non-22q11.2del patients. DGCR5 and Lnc00896 were up-regulated 9- and 5- fold in patients with lung adenocarcinomas, implying a role in cell growth and survival (Sui et al., 2016). In a separate study of lung cancer patients, DGCR5 was down-regulated, and its target, miR-1180, was increased in expression (Chen et al., 2017). Elevations in miR-1180 reduces apoptosis (Tan et al., 2016). DGCR5 also functions as a competing RNA for miR-23b, causes increases in the targets of this miRNA, PTEN and BTG1 (Xu et al., 2019). Lnc00896 is regulated by miR-139, although the importance of this remains unknown (Sui et al., 2016). DGCR11 is up-regulated 2-fold in hepatocellular cancers, again its role remaining undefined (Zhang et al., 2015). AC004471.10 has a protein coding analog in both the murine and cow genomes, termed Testes Specific Serine Kinase 2 (TSSK2), and lies downstream of TSSK1A (Liu et al., 2017). The significance of this lncRNA-protein coding region homology remains unclear. XXbac-B444P24.14 was identified in a screen for differentially regulated lncRNAs in multiple myeloma (Ronchetti et al., 2018). As with many of the annotated lncRNAs currently curated in the human genome, future studies will need to ascertain the functional contributions of those haploinsufficient due to 22q11.2del and assess the consequences of this on the clinical phenotypes.

Along with the miRNAs and lncRNAs encoded on chromosome 22q11.2 are two small nucleolar RNAs (snoRNAs), SNORA15, and SNORA77 (**Table 2**). Ranging in size from 60–300 nucleotides, snoRNAs are generated from intronic regions. They assemble with a set of proteins in the nucleolus to facilitate ribosomal RNA processing (Mcmahon et al., 2015). Both SNORA15 and SNORA77 regulate the conversion of uridines to pseudouridines to improve the folding and stabilization of rRNAs (Dupuis-Sandoval et al., 2015). The expression of SNORA15 is altered in cancer, but again how the haploinsufficiency of either of the two snoRNAs affects biological functions in the context of 22q11.2del remains an open question (Yang et al., 2017).

Taken together, it is clear that the haploinsufficiency of diverse noncoding RNAs and the extensive dysregulation of the miRNAs within this group will impact both developmental processes during embryogenesis and post-natal cardiac, immune, and neurological functions. Identifying the pathways affected by these diverse noncoding RNAs may better guide clinical management of the 22q11.2del patients.

## Diverse Genetic Polymorphisms Affecting 22q11.2del Phenotypes

Patients with both deletions and duplications of chromosome 22q11.2 and those with loss- or gain- of-function mutations in *TBX1* often have overlapping congenital problems that still vary in severity (Ensenauer et al., 2003; Yagi et al., 2003; Xu et al., 2004; Stoller and Epstein, 2005; Zweier et al., 2007; Chen et al., 2012; Reeh et al., 2014; Baldini et al., 2016). Moreover, identical mutations in *TBX1* present among several members of the same family resulted in distinct clinical phenotypes (Yagi et al., 2003). These findings further support the idea that selected genetic and/or epigenetic changes can impact the severity of the clinical complications for 22q11.2del patients (Lindsay et al., 1999; Jerome and Papaioannou, 2001; Lindsay et al., 2001; Merscher et al., 2001; Taddei et al., 2001; Xu et al., 2004; Zhang and Baldini, 2008; Chen et al., 2012; Baldini et al., 2016; Fulcoli et al., 2016). This is evident with recent studies revealing numerous genetic differences among the 22q11.2del patients that lie outside the affected locus, potentially affecting disease severity and penetrance (Guo et al., 2015; Mlynarski et al., 2015; Consortium et al., 2016; León et al., 2017). Identified by targeted exome, whole genome, and RNA sequencing strategies, SNPs, copy number variants (CNVs), DNA coding sequence differences and noncoding alterations have been discovered. For example, whole exome sequencing of 184 different 22q11.2del patients was used to identify rare deleterious SNPs (Guo et al., 2015). This screen uncovered polymorphisms in several genes selectively in those 22q11.2del patients with CHD. Several of the SNPs are in genes that regulate histone demethylation, *Jumonji Domain Containing 1C* (*JMJD1C*), *MYC Induced Nuclear Antigen* (*MINA*), and *Lysine-Specific Demethylase* (*KDM7A*). This once again reveals an epigenetic component to 22q11.2del. SNPs have also been reported in *Ras Responsive Element Binding protein 1* (*RREB1*) and *SEC24 family member C* (*SEC24C*) selectively in the 22q11.2del with CHD.

CNV analyses have revealed a connection between those 22q11.2del patients who have CHD with a duplication of *GLUT3* (also known as *SLC2A3*), located on chromosome 12p13.3 (Mlynarski et al., 2015). Its duplication is only pathogenic in conjunction with 22q11.2del (Odds ratio = 5.08) (Mlynarski et al., 2015). Of the 5 glucose transporters, GLUT3 has the highest affinity and greatest transport capacity for glucose (Simpson et al., 2008). An independent screen for CNVs in 253 22q11.2del patients identified a duplication of the first 3 exons of the *KANSL1* (chromosome 17q21.31), contributing to an increased odds ratio for CHD (OR = 2.75) (León et al., 2017). KANSL1 is part of protein complex that acetylates histones to regulate gene expression (Moreno-Igoa et al., 2015). How the duplication of 3 exons of *KANSL1* increases the likelihood of CHD remains uncertain, but a recurring theme of histone modifications in the context of 22q11.2del is evident. *KANSL1* is also linked to gene pathways regulated by miRNAs, discussed in a preceding section. In a separate screen for CNVs in miRNAs, deletions and duplications of 11 distinct miRNAs were uncovered in 22q11.2del patients, with at least 1 miRNA affected per individual (Bertini et al., 2017).

## Mouse Studies Revealing Additional Candidate Genes Influencing 22q11.2del

Complementing the human sequencing are various mouse studies suggesting additional candidate genes could affect the penetrance and/or severity of 22q11.2del. Among these are *Vascular endothelial growth factor* (*Vegf*), *Fibroblast growth factor 8* (*Fgf8*), and *Platelet derived growth factor receptor* (*Pdgfr*) (**Table 3**) (Vitelli et al., 2002; Stalmans et al., 2003; Voss et al., 2012). In mice, reductions in *Vegf* recapitulate the cardiac defects of 22q11.2del (Stalmans et al., 2003; Lambrechts et al., 2005). Mice haploinsufficient in both *Tbx1* and *Fgf8* (*Tbx1* ^+/−^ *Fgf8* ^+/−^) have a higher penetrance of aortic arch artery defects, with Tbx1 regulating *Fgf8* expression (Vitelli et al., 2002). These results led one group of investigators to speculate that *VEGF* and/or *FGF8* expression differences in humans could affect the severity of clinical phenotypes in 22q11.2del (Stalmans et al., 2003; Lambrechts et al., 2005). In one case study, a family with low levels of *VEGF* had several members diagnosed with Tetralogy of Fallot, which occurs in patients with 22q11.2del (Lambrechts et al., 2005). Another possible epigenetic regulator is *Pdgfrα*, which is expressed in neural crest cells throughout the pharyngeal region (Tallquist and Soriano, 2003; Smith and Tallquist, 2010). Its targeted elimination in mice causes very similar cardiac anomalies as reported for 22q11.2del (Tallquist and Soriano, 2003). Interestingly, *Pdgfrβ* appears to provide some functional redundancy in the absence of *Pdgfrα*, as the elimination of both receptors in neural crest cells results in a near complete penetrance of the cardiac anomalies along with a thymic aplasia (Tallquist and Soriano, 2003; Richarte et al., 2007; Smith and Tallquist, 2010). Other candidate genes identified in mouse models include *Pax1*, *Pax3*, *Rarb*, *Cyp26b1*, *Hoxa3*, *Hoxb1*, *Hoxb3*, *Hoxb4*, and *Hoxd4*, each of which is expressed either throughout the entire pharyngeal apparatus or at selected regions. Loss-of-function mutations in human *PAX1* cause otofaciocervical syndrome coupled with immunodeficiency, the latter due to the resulting thymic aplasia (Pohl et al., 2013; Paganini et al., 2017). In summary, it is likely that polymorphisms and/or mutations that impact the expression/function of many of the genes coupled to the patterning of the pharyngeal apparatus as well as neurological processes will be identified based on their capacity to modulate the clinical manifestations of 22q11.2del.

A further observation from the different mouse models is the strain dependent severity of the CHD and thymic anomalies, which reveals the effects of genetic differences (Taddei et al., 2001). Thus, the original 129SvEv background in which most of the 22q11.2del mouse models were generated has the lowest penetrance of the congenital malformations (Kimber et al., 1999; Lindsay et al., 1999; Jerome and Papaioannou, 2001; Lindsay et al., 2001; Merscher et al., 2001). Backcrossing these mice onto the C57BL/6 background yields a more penetrant phenotype, particularly in regards to the thymic hypoplasia (Taddei et al., 2001). Of relevance, this strain of mice is more sensitive to stress, as assessed by the higher corticosterone levels in the blood (Ramírez-Rosas et al., 2019). Children with 22q11.2del have higher levels of cortisol relative to age-matched controls (Jacobson et al., 2016). Elevations in cortisol can reduce T cell output from the thymus (Belkaya et al., 2011). Studies in a zebrafish model suggest elevated cortisol levels during embryogenesis are detrimental to cardiac functions (Nesan and Vijayan, 2012). The connection between stress, tissue repair and regeneration, and miRNAs is well-established, and the dysregulation of miRNAs described above will likely be impacted by the cortisol levels in 22q11.2del patients. Finally, the severity and penetrance of the congenital malformations may be related to epigenetic modifiers that affect tissue repair and regeneration during embryogenesis. Notably, the 4^th^ pharyngeal arch artery is either missing and/or poorly developed in nearly 100% of the murine 22q11.2 del embryos when assessed at embryonic e10.5 (equivalent to human embryonic week 7) (Lindsay and Baldini, 2001; Karpinski et al., 2014). However, this penetrance drops dramatically between e12.5-e18.5, such that only 30% of the 22q11.2del pups have CHD at term (Lindsay and Baldini, 2001). This indicates a regenerative or corrective process in genetically identical embryos, which could be explained by epigenetic modifiers, a possibility that requires further analysis.

## Independent Chromosomal Deletions in Relation to 22q11.2del

In an analysis of >1400 22q11.2del patients, 1% were found to have secondary mutations at other loci (Michaud et al., 1995; Mcdonald-Mcginn et al., 2013; Kruszka et al., 2017). These secondary mutations, including some affecting the presumed normal allele of chromosome 22q11.2, are resulting in dual diagnoses (Cohen et al., 2018). A few of the more prominent diagnoses include CHARGE (Coloboma, heart defects, atresia choanae, growth retardation, genital and ear anomalies) syndrome, cystic fibrosis, G6PD deficiency, 17q12 deletion syndrome, and von Willebrand disease (Michaud et al., 1995; Mcdonald-Mcginn et al., 2013; Cohen et al., 2018). Whether these secondary mutations at distinct loci influence the expression of genes on chromosome 22q11.2 has not been assessed. There are also reports of patients with 22q11.2del-like phenotypes due to chromosomal deletions at unrelated loci. Among these are microdeletions on 10p13, 4q34, and 3p12.3 (**Table 1**). Terminal deletions on the short arm of chromosome 10 cause 10p syndrome. Described in over 46 patients, the phenotypes are split into two: DiGeorge syndrome 2 (DGS2) (OMIM# 601362) and hypoparathyroidism, sensorineural deafness and renal dysplasia (HDR) (OMIM# 146255) (Van Esch et al., 2000). DGS2 results from haploinsufficiency of the more proximal region of 10p13-10p14. The resulting cardiac anomalies and thymic hypoplasia are coupled to a haploinsufficiency of *BRUNOL3* (*NAPOR, CUGBP2, ETR3*) (Lichtner et al., 2002). HDR is a separate syndrome involving deletions in the more terminal region of chromosome 10 (10p14-10pter) that result in a hemizygosity of *GATA3* (Lindstrand et al., 2010). Chromosomal deletions of the terminal end of 4q34 cause chromosome 4q syndrome, with at least one patient reported with clinical phenotypes of 22q11.2del (Cuturilo et al., 2011; Strehle et al., 2012). A 371-kb interstitial deletion of 3p12.3 was reported for a male child with a clinical presentation of 22q11.2del (Cirillo et al., 2017). Two miRNAs, miR-1243 and miR-4273 along with the *Zinc Finger Protein 717* (*ZNF717)* are affected by this deletion, resulting in DiGeorge-like syndrome and renal insufficiency. Given the clinical overlap among these different microdeletions, it is anticipated that several may affect *TBX1* expression and/or modulate histone modifications among cells differentiating within the pharyngeal region.

## Summary

Rapid advances in our understanding of the molecular mechanisms underpinning the heterogeneous clinical presentations of 22q11.2del are creating exciting new possibilities for earlier diagnosis and better treatment strategies for affected individuals. The introduction of TREC testing as part of newborn screening throughout the US and in other countries has accelerated the time when infants with 22q11.2del are diagnosed, and this will mean in more rapid and appropriate clinical interventions (Dorsey et al., 2017). Innovations in whole genome sequencing and DNA-based microarrays also make possible an accurate diagnosis of 22q11.2del in the developing fetus using maternal blood sampling (Yatsenko et al., 2015; Schmid et al., 2017). These non-invasive prenatal tests are very sensitive and have low false positive discovery rates (Ravi et al., 2018). Improvements in sequencing limited amounts of fetal DNA from maternal sampling is moving the field to 1^st^ trimester screening (Srinivasan et al., 2013; Helgeson et al., 2015) (ClinicalTrials.gov Identifier: NCT03375359). This is important because the pharyngeal defects arise during this period. Mitigating the damage to the pharyngeal region in *TBX1*-haploinsufficient embryos at this time point will definitely improve clinical outcomes. In the post-natal period, managing physiological stress should improve vulnerabilities due to miRNA dysregulation, arising from the reduced levels of *DGCR8*. The identification of small drugs able to modulate the consequences of the haploinsufficiency of both *TBX1* and *DGCR8* suggest new strategies for reducing the clinical penetrance and severity of 22q11.2del (Barr et al., 2015; Fulcoli et al., 2016). In summary, DNA and RNA sequencing approaches are unveiling new genetic and epigenetic modifiers among the 22q11.2del cohort that have a significant impact on disease penetrance and severity. Identifying and understanding these genetic and epigenetic regulators in an individualized manner for each 22q11.2del patient will direct future care in order to minimize disease severity.

## Author Contributions

All authors, QD, MM, and NO contributed to the review of the literature, the writing of the manuscript, and the preparation of data in tables and figures.

## Funding

Our work was supported, in part, by grants from the National Institutes of Health R01 (R01 AI114523, R21 AI144140 NO), Beecherl funds from the Department of Immunology at UT Southwestern Medical Center (NO), and the Jeffrey Modell Foundation (MM).

## Conflict of Interest

The authors declare that the research was conducted in the absence of any commercial or financial relationships that could be construed as a potential conflict of interest.
